# What Predicts Different Kinds of Nonadherent Behavior in Elderly People With Parkinson's Disease?

**DOI:** 10.3389/fmed.2020.00103

**Published:** 2020-03-25

**Authors:** Sarah Mendorf, Otto W. Witte, Julian Grosskreutz, Hannah M. Zipprich, Tino Prell

**Affiliations:** ^1^Department of Neurology, Jena University Hospital, Jena, Germany; ^2^Center for Healthy Ageing, Jena University Hospital, Jena, Germany

**Keywords:** nonadherence, Parkinson's disease, unified Parkinson's disease rating scale, German stendal adherence with medication score, motor impairment

## Abstract

**Background:** Detailed knowledge about nonadherence to medication could improve medical care in elderly patients. We aimed to explore patterns and reasons for nonadherence in people with Parkinson's disease (PD) aged 60 years and older.

**Methods:** Detailed clinical data and adherence (German Stendal Adherence with Medication Score) were assessed in 230 patients with PD (without dementia). Descriptive statistics were used to study reasons for nonadherence in detail, and general linear models were used to study associations between clusters of nonadherence and clinical parameters.

**Results:** Overall, 14.2% (*n* = 32) of the patients were fully adherent, 66.8% (*n* = 151) were moderately nonadherent, and 19.0% (*n* = 43) showed clinically meaningful nonadherence. In the multivariable analysis, nonadherence was associated with a lower education level, higher motor impairment in activities of daily living, higher number of medications per day, and motor complications of PD. Three clusters of nonadherence were observed: 59 (30.4%) patients reported intentional nonadherence by medication modification; in 72 (37.1%) patients, nonadherence was associated with forgetting to take medication; and 63 (32.5%) patients had poor knowledge about the prescribed medication. A lower education level was mainly associated with modification of medication and poorer knowledge about prescribed medication, but not with forgetting to take medication. Patients with motor complications, which frequently occur in those with advanced disease stages, tend to be intentionally nonadherent by modifying their prescribed medication. Increased motor problems and a higher total number of drugs per day were associated with less knowledge about the names, reasons, and dosages of their prescribed medication.

**Conclusions:** Elderly patients with PD report many reasons for intentional and non-intentional nonadherence. Understanding the impact of clinical parameters on different patterns of nonadherence may facilitate tailoring of interventions and counseling to improve outcomes.

## Introduction

Parkinson's disease (PD) is the second most common neurodegenerative disease (1). Both the prevalence and incidence of Parkinson's disease increase continuously with age. About 0.5% of patients in the age group 60–69 years suffer from PD in western industrial nations, while about 1.6% of patients in the age group 70–79 years are already affected (1). It is well known that nonadherence to medication is a major issue in chronic disorders such as Parkinson's disease (PD) (2–6). The direct and indirect costs of nonadherence are enormous, and nonadherence contributes to poor outcomes and a lower quality of life (7–10). There are various reasons as to why people do not or cannot follow the instructions they are given for prescribed treatments. Systematically, the factors associated with adherence to medications can be divided into patient characteristics, disease-related factors, financial and health system barriers, patient–provider relationship factors, and treatment-related factors (10, 11). Patient-related factors can be further categorized as intentional (when the patient purposefully decides not to adhere to the recommended treatment) or unintentional (when the patient cannot follow the recommendations). Several studies and reviews explored epidemiological and clinical factors associated with nonadherence to medication in PD, such as younger age, education level, marital status, mood disorders, cognition, disease duration, and regimen complexity (2, 4, 6, 11, 12). However, the sample size in many studies was low (2), or, as in a large study by Valldeoriola et al., cognition as an important cofactor was not assessed in detail (it was graded according to a four-point scale). Moreover, patients with cognitive deficits were not excluded from the analysis, which may influence the validity of a self-reported adherence outcome measure (13). Therefore, replication of the observed associations between clinical parameters and nonadherence in larger, well characterized cohorts is required.

While many studies analyzed the relationship between clinical factors and the presence or absence of nonadherence, little is known about how distinct clinical factors (e.g., degree of motor disability and disease duration) modulate *the type of* nonadherent behavior. For example, do patients with higher functional impairment more frequently modify or forget their prescribed medication than those with better motor performance? (14).

Therefore, our study had the following aims:

- To describe common self-reported reasons for nonadherence in PD.
- To replicate the associations between different degrees of nonadherence and PD-specific clinical parameters.
- To explore the impact of PD-specific clinical parameters on distinct clusters/reasons of nonadherence.

## Methods

### Participants and Assessments

This observational study was approved by the local ethics committee of the Jena University Hospital (4572-10/15). All subjects gave written informed consent in accordance with the Declaration of Helsinki. Patients with PD were consecutively recruited between June 2017 and December 2018 from the outpatient clinic and the ward of the Department of Neurology of the Jena University Hospital, Germany. The inclusion criteria were PD diagnosis according to Movement Disorder Society (MDS) diagnostic criteria and stability under dopaminergic treatment. The exclusion criteria were cerebrovascular disorders, delirium, deep brain stimulation, enteral levodopa/carbidopa infusion, apomorphine infusion, and inability to complete a questionnaire. All tests were conducted during the medication ON phase. The demographic data collected included age, gender, marital status (single/divorced/widowed or married), and level of education (high: German Abitur or University; medium: German Realschule or General Certificate of Secondary Education; low: German Hauptschule or no school). Several clinical parameters were recorded: the total daily number of medications administered in any pharmaceutical form, the levodopa equivalent daily dose (15), the MDS-sponsored revision of the Unified Parkinson's Disease Rating Scale (MDS-UPDRS), the revised non-motor symptoms questionnaire (NMS-Quest), and Hoehn & Yahr staging. The MDS-UPDRS covers the patient's motor experience of daily living (II), clinician's motor examination (III), and motor complications (IV). Cognition was assessed by using the Montreal Cognitive Assessment (MoCa) (16). Beck's depression inventory II (BDI) was used to quantify depressive mood. Adherence was assessed by using the self-reported German Stendal Adherence with Medication Score (SAMS). It includes 18 questions forming a cumulative scale (0–72) in which 0 indicates complete adherence and 72 indicates complete nonadherence (17). It allows the assessments of three common reasons/cluster of nonadherence: modifications of medication, forgetting to take the medication, and lack of knowledge about medications (14). The full SAMS is available online (CC BY NC 3.0 license): https://data.mendeley.com/datasets/ny2krr3vgg/1 (18). It was so far not explicitly used in patients with PD, but in a mixed cohort of patients with different neurological disorders (also including people with PD) (14).

The total number of patients recruited for the study was 300. Seventy-four cases had incomplete or missing data or PD dementia (MoCa < 21) (16) and were excluded. Therefore, 226 patients were included in the final analyses.

### Statistical Analysis

The SPSS statistical computer package (version 25.0; IBM Corporation, USA) was used for all statistical analyses. Values are given as mean and standard deviation or median and interquartile range. Categorical variables are presented as numbers or percentages. Although the validity of the historical 80% threshold remains uncertain (19), it is generally considered that suboptimal adherence becomes clinically significant when <80% of the prescribed medication is taken (20, 21). This leads to a study- and sample-specific SAMS cut-off of 13 points for clinically meaningful/significant nonadherence. The patients were then categorized into (1) fully adherent (SAMS = 0), (2) moderately nonadherent (SAMS 1–12.9), and (3) nonadherent (SAMS ≥ 13).

Based on our previous work, each patient was categorized into one cluster of nonadherence: modifications, lack of knowledge, and forgetfulness (14). In the cluster “modifications,” for example, medications were adjusted by patients without consulting their doctor on either experiencing side effects or improvement in health. The cluster “missing knowledge” involved patients who were unaware of the purpose of their medications and/or dosages. The cluster “forgetfulness” included patients who frequently forgot to take their medications. For this purpose, a principal component analysis (PCA) (eigenvalues >1 and varimax rotation) was used to reduce the 18 SAMS items into factors representing these different nonadherence clusters. The structure of the SAMS (items 1–18) was examined by using an exploratory factor analysis. Both the Bartlett test (*p* < 0.001) and the Kaiser–Meyer–Olkin Measure of Sampling Adequacy (0.80) indicated that the variables were suitable for factor analysis. Three items, item 4 (Do you take your medication regularly?), item 7 (Are you untroubled about taking the medication?) and item 18 (If you take medication from a syringe or in a weekly tablet, have you ever forgotten it?), exhibited a low communality score and were removed from the analysis. A three-factor solution was chosen based on the scree plot, which accounts for 62.0% of the variance. The SAMS items belonging to each factor are displayed in Table 1. For every patient, the regression coefficient for each PCA factor was calculated. Descriptive statistics were used to describe the reasons for nonadherence in each cluster in detail. A general liner model (stepwise forward, Akaike information criterion) was calculated to study the association between a set of clinical variables and overall adherence (SAMS total). The set of clinical variables (independent variables) was derived from the literature and included gender, education level, marital status, total number of prescribed drugs, LEDD, H&Y stage, MDS-UPDRS II, III, and IV, NMS-Quest, MoCa, BDI, and disease duration (2–4, 6, 11–13, 20, 22, 23). The clinical variables which were found to be significantly associated with nonadherence were then entered as independent variables into three additional linear models with (1) degree of modification (regression coefficients for the cluster “modification”), (2) degree of missing knowledge (regression coefficients for the cluster “missing knowledge”), and (3) degree of forgetfulness (regression coefficients for the cluster “forgetfulness”) as dependent variables (stepwise forward, Akaike information criterion). The threshold for statistical significance was set at *p* < 0.05.

**Table 1:** Principal component analysis.

	**Factor 1 Modifications**	**Factor 2 Missing knowledge**	**Factor 3 Forgetfulness**
12 If you think you have side effects due to of the medications (such as tremors, nausea etc.), do you not take the medication for a while, i.e., take a break?	0.830		
11 If you think you have side effects due to of the medications (such as tremors, nausea etc.), do you reduce the dose without consulting a doctor?	0.765		
13 If you feel you have to take too many, do you stop taking those medications you consider to be less important than the others without consulting your doctor?	0.762		
10 Do you take any wrong or other/unprescribed medications (such as those of your partner)?	0.709		
9 Do you stop taking your medication if you sometimes feel worse after taking the medication?	0.698		
17 Do you deliberately not take medications you do not consider important, but take the rest?	0.637		
8 Do you stop taking your medication when you feel better?	0.613		
2 Do you know the dosages of your medication?		0.887	
1 Do you know the reason for taking your medication?		0.855	
5 Do you know the names of medications you are taking?		0.805	
3 Are you familiar with the timing for taking the medication?		0.775	
15 If you forget or omit your medication, do you forget it at noon?			0.887
16 If you forget or omit your medication, do you forget it in the evening?			0.832
14 If you forget or omit your medication, do you forget it in the morning?			0.723
6 Do you forget to take your medication?			0.623

## Results

### Prevalence of Nonadherence and Its Association With Clinical Parameters

**Figure 1:**
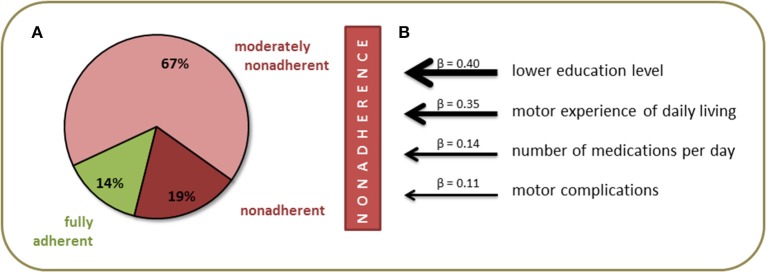
Prevalence **(A)** and predictors **(B)** of nonadherence in people with Parkinson's disease.

The final sample included 226 patients with PD [95 (41.3%) female] with a mean age of 71.1 ± 7.9 years. The majority of patients was married and had completed middle or high school education. Detailed clinical data are provided in Table 2. The mean total SAMS was 7.4 ± 7.7 points. According to the SAMS, 14.2% (*n* = 32) of the patients were fully adherent (SAMS = 0), 66.8% (*n* = 151) were moderately nonadherent (SAMS 1–12.9), and 19.0% (*n* = 43) showed clinically meaningful nonadherence (SAMS ≥ 13) (Figure 1A). In the multivariable analysis, nonadherence was associated with a lower education level (β = 0.40, *p* = 0.002), higher rate of motor impairment in activities of daily living (MDS-UPDRS II, β = 0.35, *p* = 0.004), higher number of medications per day (β = 0.14, *p* = 0.066), and higher rate of motor complications of PD (MDS-UPDRS IV, β = 0.11, *p* = 0.104) [*F*_(4, 225)_ = 7.2, *p* < 0.001] (Figure 1B).

**Table 2:** Demographic and clinical characteristics.

		***n***	**%**
Sex	Female	131	58.0
	Male	95	42.0
Marital status	Married	161	71.2
	Single, divorced, widowed, separated	61	27.0
	Missing data	4	1.8
Education level	High	90	39.8
	Middle	84	37.2
	Low	46	20.4
	Missing data	6	2.6
		**Mean**	***SD***
Age (years)		70.9	7.9
SAMS total		7.4	7.7
Number of drugs per day		5.9	3.3
Disease duration (years)		9.4	6.9
Levodopa equivalent daily dose (mg)		681	542
MDS-UPDRS II		18.2	9.03
MDS-UPDRS III		26.9	15.3
MDS-UPDRS IV		4.2	4.1
Non-motor symptoms questionnaire total		11.3	5.4
Becks Depression Inventory II total		12.7	8.6
Montreal Cognitive Assessment total		24.7	2.9
		**Median**	**IQR**
Hoehn & Yahr stage		3	1

*MDS-UPDRS, Movement Disorder Society-sponsored revision of the Unified Parkinson's Disease Rating Scale; SAMS, German Stendal Adherence with Medication Score; Education levels: low (no school, German Hauptschule), middle (General Certificate of Secondary Education, German Realschule), high (German Abitur or University). IQR, interquartile range*.

### Reasons for Nonadherence

**Figure 2:**
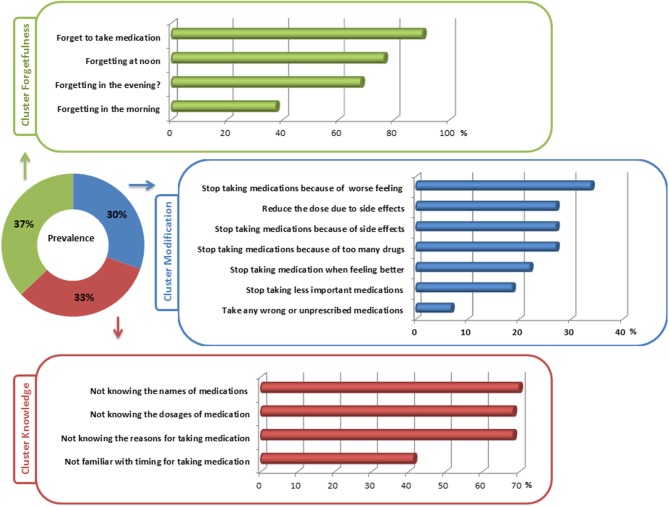
Detailed reasons for nonadherence in the three clusters of nonadherence.

In the nonadherent group (all with SAMS >1), 59 (30.4%) patients reported intentional nonadherence by modification of medication. In this cluster, patients most frequently reported stopping medication because of side effects or feeling worse after taking the medication. Interestingly, 22% discontinued medication when they felt better and 19% stopped taking medication that they considered to be less important (Figure 2). Nonadherence associated with forgetfulness was found in 72 (37.1%) of PD patients. Of note, most patients in this cluster forgot to take their medication at noon. Forgetting to take medication was reported to be less in the morning (Figure 2). In 63 (32.5%) patients, nonadherence was caused by lack of knowledge about the prescribed medication. The majority did not know the exact names, the reasons for, and the dosages of their prescribed medications (Figure 2).

### Patterns of Nonadherence and Their Association With Clinical Parameters

In the multivariable analyses, the cluster “modification” was associated with a lower education level (β = 0.63, *p* = 0.05) and a higher rate of motor complications (MDS-UPDRS IV, β = 0.37, *p* = 0.14) [*F*_(2, 147)_ = 3.1, *p* = 0.049]. The cluster “missing knowledge” was associated with a higher total number of drugs per day (β = 0.37, *p* = 0.019), a lower education level (β = 0.35, *p* = 0.022), and higher motor impairment (MDS-UPDRS II, β = 0.28, *p* = 0.028) [*F*_(4, 147)_ = 5.8, *p* = 0.001]. Forgetting to take medication was not significantly associated with any of the clinical parameters.

## Discussion

### Prevalence of Nonadherence and Its Association With Clinical Factors

Medication adherence in PD, defined as taking >80% of the prescribed dose, ranged widely from 33 to 97.7% in previous studies (11). The prevalence mainly depends on the method used to assess adherence and the study design. Measures of adherence can be classified as direct and indirect. Direct methods (e.g., measurements of the drug or its metabolite concentration in body fluids) are accurate, but have several drawbacks (expensive, improvement of adherence before the upcoming tests, individual metabolic rates, drug-drug or drug-food interactions) (24). Their low cost, simplicity, and real-time feedback make indirect methods (self-reports, interviews) interesting tools to identify individual patient concerns. Estimates of non-adherence prevalence in PD range from 15 to 20% by self-report, to 67% and higher in studies using pharmacy refill data and pill counts (22). Given that self-reports overestimate adherence compared against electronic monitoring, in clinical practice, when self-reports are less than 80%, adherence is extremely likely to be suboptimum (25). However, only self-reports allow a statement about the personal reasons of nonadherence. The prevalence of clinical significant nonadherence observed in our cohort was comparable to that of other studies using self-report in PD patients (2, 22). However, it should be noted that many studies only made a general distinction between adherent/nonadherent. In order to better reflect the broad spectrum of adherence, we have made a classification into three different degrees of adherence (fully, moderate, clinically significant). This shows that nonadherent behavior is very common in elderly people with PD, but it does not always have to be associated with clinical significance.

We found a higher degree of nonadherence in patients with a lower education level, higher motor impairment, a higher number of medications per day, and more motor complications of PD. Associations between a poor clinical state, polypharmacy, or a higher levodopa equivalent dose and nonadherence were reported previously (2–4, 12, 13, 26). Several factors formerly reported to be associated with nonadherence in PD, namely depression, cognitive state, younger age, and disease duration, were not significant predictors of nonadherence in our cohort of elderly PD patients without dementia (2). This requires further consideration. The lack of an association with age may be explained by our focus on elderly subjects and the exclusion of young-onset PD. In contrast, studies which reported that increased age was associated with better overall or timing adherence used electronically monitored bottles to assess adherence (3, 4). These studies are not comparable to our study with a self-report outcome measure, although our sample size was higher. Therefore, we need more sufficient data to substantiate the relationship between adherence and age. We also did not observe a significant association between cognitive disturbances and the overall adherence in the SAMS. The association between cognition and adherence is complex and not yet fully understood (27). While some studies found that cognitive impairment is, in general, a major predictor of nonadherence (28, 29), a recent meta-analysis found no association between cognitive impairment and medication nonadherence in post-stroke patients (23). The Adheson study showed that in relation to the Morisky–Green test, patients with cognitive deterioration were 2.1 times more likely to take the treatment incorrectly (13). Possibly, we did not find an association between adherence and cognition because our study was restricted to PD patients without dementia. This is because we assumed that cognitive deficits may influence the validity of a self-report adherence measure. We also did not observe an association between disease duration and nonadherence. However, we observed an association between motor complications (dyskinesia and dystonia) and nonadherence. Considering that motor complications usually occur in advanced PD disease stages, we conclude that functional impairments are more relevant to nonadherence than disease duration *per se*.

### Self-Reported Reasons for Nonadherence

The use of a comprehensive adherence questionnaire allowed us to gain deeper insight into the reasons for and patterns of nonadherence in PD. A number of studies have shown that intentional nonadherence comprises approximately 50% of all nonadherent behaviors observed among those over the age of 65 years (30). The most common reasons given by our patients for modification of prescribed medication were side effects or feeling worse after taking the medication. However, a relevant proportion of patients do not stop taking medications because they feel worse but because they feel better. Given the plethora of reasons for intentional nonadherence, an individual approach is necessary to positively modulate adherence in elderly people with PD (31, 32). Because many people discontinue medication because of side effects, comprehensive education on side effects and what to do in case of side effects is recommended for every patient. To avoid discontinuation of medication when people feel better, patients must be constantly reminded of the importance of taking medications on a long-term basis in order to positively influence PD symptoms and the course of the disease in the long term.

Forgetting to take medication was the main reason for nonadherence in 37% of patients. Therefore, it is not surprising that interventions to increase adherence which mainly work with reminders are not useful to enhance adherence in general (33). Interestingly, in our study, most patients forgot to take their medication at noon. Therefore, to give long-acting medications, preferably in the morning, seems to be a sensible way to improve adherence.

Lack of knowledge as the main reason for nonadherence was reported in 32.5% of patients. Many patients have problems with the timing of medication (4, 34). However, given the progressive nature of the disease and the occurrence of motor fluctuations during the disease course, correct timing is essential for efficient control of motor symptoms. In line with a former study in older subjects, a relevant proportion of our patients did not exactly know the names, reasons, and dosages of all their prescribed medications (35). Moreover, in other conditions and cohorts, lack of knowledge was found to be a critical determinant of nonadherence independent of education (36, 37).

### Factors Associated With Distinct Patterns of Nonadherence

We found that the different clusters of nonadherence were differentially influenced by clinical parameters. A lower education level was mainly associated with modification of medication and poorer knowledge about prescribed medication, but not with forgetting to take medication. We also found that patients with motor complications (higher MDS-UPDRS IV), which frequently occur in advanced disease stages, tended to demonstrate intentional nonadherence by modifying their prescribed medication. This was probably the result of patients' desire to exert control over the treatment and its effects on their bodies (31). Higher motor problems and the consequent higher total number of drugs per day were associated with less knowledge about the names, reasons, and dosages of their prescribed medication.

The study is not free of limitations. Because we were interested in patient-reported and personal-related factors of nonadherence, we did not compare the SAMS with other adherence measures such as electronic monitoring. While this did not affect the results regarding reasons for nonadherence, our estimated prevalence of nonadherence must be considered with caution. This is because self-reports overestimate adherence compared with electronic monitoring (25). Although we included the main known predictors of nonadherence in PD, we were not able to control for all factors influencing adherence (e.g., presence of caregivers, comorbidities etc.). Moreover, our results were restricted to elderly PD patients without PD dementia. Therefore, these results cannot be generalized to PD patients with significant cognitive decline.

## Conclusions

Elderly PD patients report many reasons for intentional and non-intentional nonadherence. Forgetting to take medication is common, but it is not the main reason for nonadherence. Several clinical factors were found to be relevant to the question of whether a patient tends to modify or forget medication or has poor knowledge about the prescribed medication. This detailed knowledge about personal factors of nonadherence may positively influence the management of nonadherence in medical care and pave the way toward tailored, patient-centered, theory-driven interventions to improve adherence.

## Data Availability Statement

The datasets generated for this study are available on request to the corresponding author.

## Ethics Statement

The studies involving human participants were reviewed and approved by Ethics committee of the Jena University, Jena, Germany. The patients/participants provided their written informed consent to participate in this study.

## Author Contributions

TP: design of the study. SM, HZ, and TP: collection of data. JG: data management. SM, TP, and HZ: analysis. SM: writing the paper. OW and TP: critical revision of the article.

### Conflict of Interest

The authors declare that the research was conducted in the absence of any commercial or financial relationships that could be construed as a potential conflict of interest.

## Abbreviations

BDI: Becks Depression Inventory
H&Y: Hoehn & Yahr
MDS-UPDRS: Movement Disorder Society-sponsored revision of the Unified Parkinson's Disease Rating Scale
NMS-Quest: non-motor symptoms questionnaire
PD: Parkinson's Disease
SAMS: Stendal Adherence with Medication Score.
